# A multi-generational risk assessment of Cry1F on the non-target soil organism *Folsomia candida* (Collembola) based on whole transcriptome profiling

**DOI:** 10.7717/peerj.6924

**Published:** 2019-05-10

**Authors:** Cheng-Wang Huang, Wan-Jun Chen, Xin Ke, Yunhe Li, Yun-Xia Luan

**Affiliations:** 1 Key Laboratory of Insect Developmental and Evolutionary Biology, Shanghai Institute of Plant Physiology and Ecology, Chinese Academy of Sciences, Shanghai, China; 2 State Key Laboratory for Biology of Plant Diseases and Insect Pests, Institute of Plant Protection, Chinese Academy of Agricultural Sciences, Beijing, China; 3 Guangdong Provincial Key Laboratory of Insect Developmental Biology and Applied Technology, Institute of Insect Science and Technology, School of Life Sciences, South China Normal University, Guangzhou, China

**Keywords:** Environmental risk assessment, Cry1F, Collembolan, Consecutive generations, Transcriptome analysis

## Abstract

The *Bacillus thuringiensis* toxin Cry1F has been used to develop insect-resistant genetically engineered crops. There has been great interest in evaluating its potential risk to non-target organisms (NTOs). However, the majority of previous risk assessments only examined one generation of NTOs using several physiological indicators, which cannot comprehensively detect some potential sub-lethal effects at the molecular level. In this study, we conducted a laboratory-based, multi-generational risk assessment of Cry1F for the collembolan *Folsomia candida*, an important representative of soil arthropods in terms of survival, reproduction, and differentially expressed genes (DEGs) identified from whole transcriptome profiles. Our results demonstrated that Cry1F was continuously ingested by collembolans over three consecutive generations, but it did not affect the survival or reproduction of *F. candida*. There were no significant differences in the global gene expression between *F. candida*—fed diets with and without Cry1F, and no consistent co-expressed DEGs over three generations. In addition, Cry1F did not obviously alter the expression profiles of seven sensitive biological markers. Our composite data indicates that Cry1F had no long-term harmful effects on collembolan *F. candida*.

## Introduction

Over the past 20 years, genetically engineered crops have been widely planted throughout the world. The most common of these are insect-resistant genetically engineered (IRGE) crops, including IRGE cotton, maize, and others (International Service for the Acquisition of Agri-biotech Applications (ISAAA), 2016). All the current commercialized IRGE crops express *cry* or *vip* genes derived from the bacterium *Bacillus thuringiensis*, and these genes encode insecticidal proteins targeting lepidopteran or coleopteran insect pests (Bravo et al., 2011; Liu et al., 2016; Zhao et al., 2016). Although the planting of IRGE crops will greatly reduce the use of broad-spectrum insecticides, the potential risks of IRGE crops to the environment and human health must be assessed prior to their commercialization. One of the assessments is their potential negative effects on non-target organisms (NTOs) within agroecosystems based on a tiered approach from laboratory to field (European Food Safety Authority (EFSA), 2010; Li et al., 2014; Romeis et al., 2008; Shelton, Zhao & Roush, 2002; Svobodová et al., 2015). To date, substantial laboratory and field tests have been conducted with many different NTOs belonging to a range of orders, for example, ladybird beetles, honeybees, parasitic wasps, collembolans, earthworm, and zebrafish, covering different functional groups, including herbivores, predators, parasitoids, and detritivores (Gao et al., 2018; ILSI (International Life Sciences Institute) Research Foundation, 2013; Jia et al., 2016; Marques et al., 2018; Neher, Muthumbi & Dively, 2014; Tian et al., 2014; Svobodová et al., 2015).

Collembolans are an important group of detritivore soil arthropods because of their large numbers in the soil (10^4^–10^5^ m^−2^), great contribution to the decomposition of plant residues, and sensitivity to many soil pollutants (Buch et al., 2016; Chen et al., 2015; Hopkin, 1997; Zortéa et al., 2015). They are commonly exposed to *B. thuringiensis* toxins when IRGE crop residues decompose in agricultural lands (Li et al., 2007; Valldor et al., 2015). As a standard test organism that is often used for toxicological, biosafety, and environmental assessment, the parthenogenetic collembolan *Folsomia candida* is also an important NTO for the risk assessment of IRGE crops. Most previous risk assessments found no harmful effects of various *B. thuringiensis* toxins on collembolans, regardless of whether the testing was done in the laboratory or field (ILSI Research Foundation, 2013; Marques et al., 2018; Sims & Martin, 1997; Yang et al., 2015, 2018; Zhang et al., 2017a). However, most previous risk assessments have been limited to several physiological indicators, for example, survival, reproduction, and some enzyme activities, which may not detect potential weak effects. In addition, most studies only examined one generation of *F. candida*, and the potential risk to the collembolan progeny may be missing.

As the field case always involves “multi-generational” exposure, some potential sub-lethal effects of an environmental factor may cumulate over time and become apparent only in a living organism after exposure over multiple generations. Noordhoek et al. (2018) found a metal-based nanomaterial tungsten carbide-cobalt affected the reproduction and survival of *F. candida* from the third generation. By using multi-generational laboratory risk assessments, Bakonyi et al. (2011) and Szabó, Seres & Bakonyi (2017) found that *F. candida* that were fed Cry1Ab expressing maize leaves had some alterations in life-history traits and reproduction. To our knowledge, these are the only studies that have evaluated the long-term potential risk of *B. thuringiensis* toxins to collembolans. However, it is difficult to tell whether the effects were caused by the *B. thuringiensis* toxin or other components of the maize leaves and additional studies are needed.

In assessing the effects of *B. thuringiensis* toxins on collembolans, most previous studies have used some physiological indices (survival rates, reproductive rates, developmental duration, etc.) or biological markers (enzyme activity, midgut bacterial diversity, etc.). These traditional methods of risk assessment can reflect the life status of collembolans, but may not detect slight changes at the molecular level. By using microarray and qPCR, Yuan et al. (2014) found 11 gene transcripts of *F. candida* that responded to the *B. thuringiensis* proteins (Cry1Ab and Cry1Ac). RNA sequencing (RNA-Seq), which is different from microarray and qPCR, can provide a whole transcriptome profile of NTOs to check stress-responsive changes in the gene expression, which makes it possible to evaluate unknown effects, and helps us find the potential non-lethal chronic toxicity of *B. thuringiensis*. Based on RNA-Seq data, Zhang et al. (2017b) annotated 32,268 unigenes of the Chinese green mussel (*Perna viridis*) and identified 9,048 differentially expressed genes (DEGs) between exposed and non-exposed groups to cadmium, suggesting a sensitive response of the mussel transcriptome to cadmium. Noordhoek et al. (2018) studied phenotypic effects and transcriptional responses in *F. candida* exposed to nanomaterials and detected physiological alterations cumulating from one generation to the next based on gene expression assays but without visible phenotypic variables.

Cry1F has been transferred to maize, cotton, and others, and previous studies have found no harmful effects of Cry1F on some tested NTOs, including *F. candida* (Higgins et al., 2009; ILSI Research Foundation, 2013; Kim et al., 2012; Marques et al., 2018; Tian et al., 2014). However, its risk to collembolans has not been evaluated as comprehensively as other Cry proteins such as Cry1Ab, Cry1Ac, Cry2A, and Cry1C. In this study, we performed a dietary exposure experiment (DEE) with *F. candida* that was fed an artificial diet containing or excluding purified Cry1F protein for three consecutive generations. We compared the effects of these two diets on the survival, reproduction, and transcriptome changes of *F. candida* for each generation. To our knowledge, this is the first multi-generational laboratory risk assessment of Cry1F on *F. candida*, and also the first attempt to use RNA-Seq to evaluate the impact of a *B. thuringiensis* toxin on the gene expression of *F. candida*.

## Materials and Methods

### Test organism

**Figure 1:**
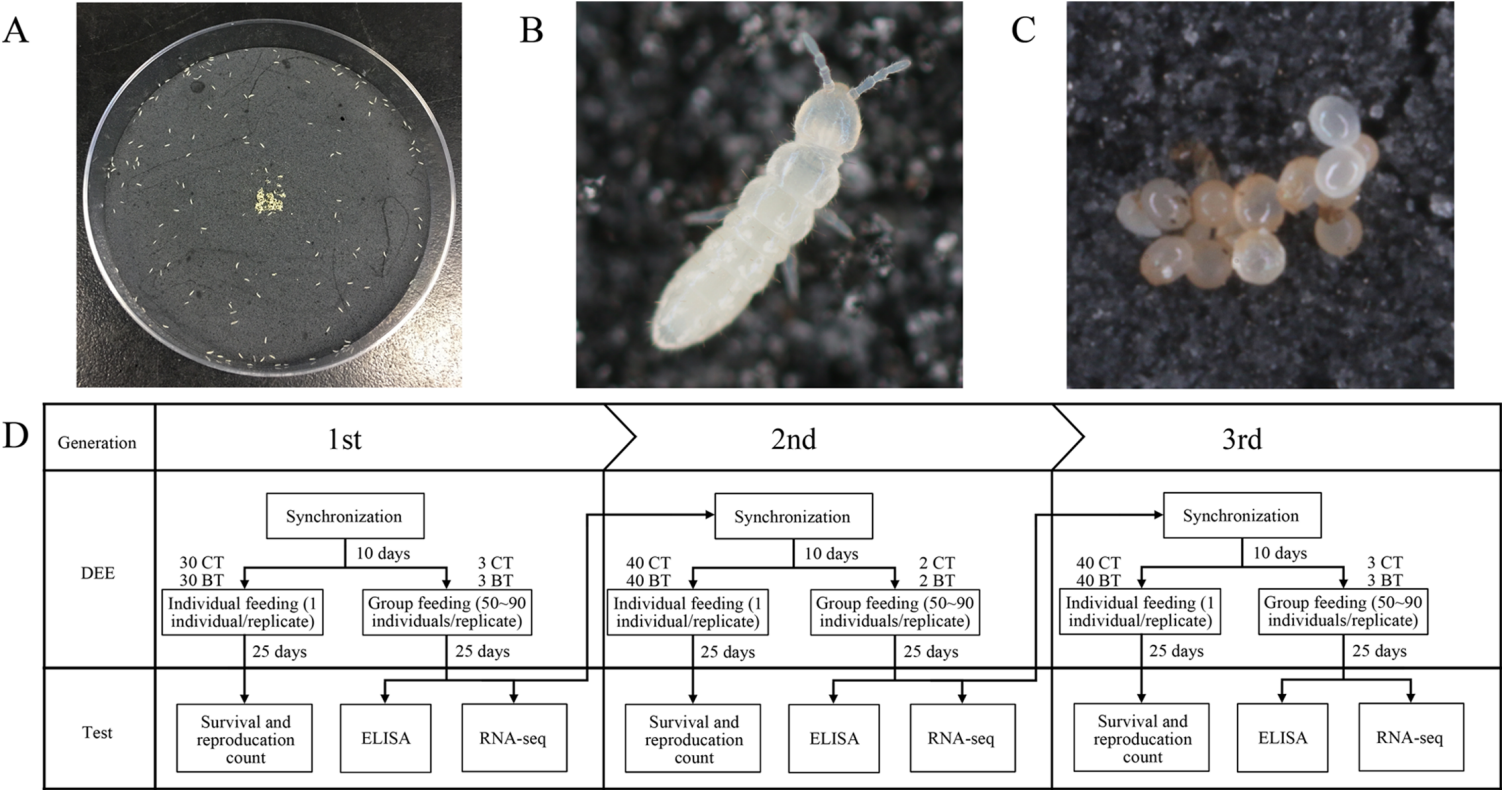
Culture of *Folsomia candida* and the flow chart of the experiment. (A) *F. candida* reared in a Petri dish with baker’s yeast as food. (B) A living individual of *F. candida*. (C) A cluster of *F. candida* eggs. (D) The flow chart of the dietary exposure experiment over three consecutive generations. The CT set of collembolans was fed yeast powder without Cry1F, and the BT set was fed yeast powder with Cry1F. In each generation, both CT and BT sets were fed and evaluated individually (with 30 or 40 replicates) or in groups of 60–80 individuals (with two or three replicates).

The Danish strain of the parthenogenetic *F. candida* Willem, 1902, was originally obtained from Aarhus University, Denmark, and has been cultured in our laboratory for over 10 years. *F. candida* is usually fed granulated dried baker’s yeast (Hebei Mauri Foods Co., Ltd., Zhangjiakou, China) and was reared in the Petri dishes (90 × 18 mm) containing a five to seven mm layer of a solidified mixture of plaster of Paris and activated charcoal (9:1 wt/wt, dissolved in distilled water, with about 270 ml of water/500 g of mixed powder) (Figs. 1A–1C). The baker’s yeast was placed on the surface of the plaster and was renewed weekly to reduce the growth of other fungi. Distilled water was added to the base as needed such that free water was present in the plaster pores but did not form a film on the plaster surface. These dishes with *F. candida* were kept in an artificial climate chamber in total darkness (20 ± 1 °C, 80% relative humidity).

### Dietary exposure experiment

The artificial diet containing Cry1F (BT diet) was prepared as follows: two mg of purified Cry1F protein (Envirologix Inc., Portland, ME, USA) and four g of baker’s yeast granules were dissolved in 10 ml of distilled water; the preparation was fully mixed and then transferred into a plastic container. The final concentration of Cry1F protein in the BT diet was 500 μg/g, which is much higher than the Cry1F protein concentrations estimated in IRGE crops (less than 50 μg/g, ILSI Research Foundation, 2013), as well as than the EC_50_ values of Cry1F against lepidopteran pests *Chilo suppressalis* (6 μg/g) and larvae of *Bombyx mori* (136 ng/g) (Jiao et al., 2016). After 27 h of lyophilization, the BT diet was ground into powder and stored at −70 °C. As the control, baker’s yeast granules without Cry1F (CT diet) were prepared in the same way. We also tried a positive control by adding the insecticidal compounds (potassium arsenate) into the baker’s yeast granules, but it significantly affected the survival and the development of *F. candida*, which hampered the performance of the consecutive multi-generational exposure experiment. Therefore, we didn’t perform the positive control in our multi-generational DEE. However, the concentration of Cry1F in the BT diet was high enough to validate the test of Cry1F to *F. candida*.

Based on the Organization for Economic Cooperation and Development test protocol (Organisation for Economic Cooperation and Development (OECD), 2016), we designed a multi-generational DEE on *F. candida* (Fig. 1D). During the experiment, all *F. candida* were cultured in the same artificial climate chamber with the culture conditions mentioned above and were synchronized to the same stage (neonates) at the start of each generation in the experiment.

First, we transferred 80–100 randomly selected *F. candida* adults (about 4–6 weeks old) to a new Petri dish. After 72 h of oviposition, all adults were removed. The eggs began to hatch 10 days later. After 24 h of hatching, we selected synchronized neonates. They were transferred to a new Petri dish and were fed with pure baker’s yeast for 10 days. Next, all juveniles were separated into two sets: the BT set were fed BT diet, and the CT set were fed CT diet. Each set was done with individually- and group-reared collembolans.

In the first-generation DEE, 60 juveniles were transferred to 60 small plastic Petri dishes (55 × 14 mm, plaster height: 5 mm), respectively, as individually-reared collembolans. A total of 30 were fed the BT diet, and 30 were fed the CT diet. The remaining juveniles were evenly distributed into six plastic Petri dishes (85 × 16 mm, plaster height: 5 mm), with 60–80 collembolans per dish. Three of them were fed the BT diet and the other three were fed the CT diet. All diets were renewed every 2 days to avoid Cry1F protein degradation and the growth of other fungi.

The first generation was assessed after 25 days of DEE, which is 35 days from hatching. We choose this time point to ensure that *F. candida* had sufficient exposure to Cry1F, and had laid one to two batches of eggs, but with only one batch of eggs hatching. For the individually-reared collembolans, the numbers of adults and juveniles in each treatment were counted to calculate the survival and reproduction rates. For the collembolans fed in groups, about 20 individuals per replicate were randomly collected for enzyme-linked immunosorbent assay (ELISA, see section *Enzyme-linked immunosorbent assay* below), and 25–35 individuals per replicate were collected for RNA-Seq (see section *RNA extraction, sequencing, and unigene annotation* below).

The remaining collembolans fed in groups of CT and BT sets were used for synchronization of the second generation. To maintain the same experimental conditions, the CT and BT sets were synchronized independently, and their neonates were fed the CT diet or BT diet directly after birth. After 10 days, the second-generation DEE began and was conducted in the same manner as the first-generation DEE except that there were 40 replicates per set for individually-reared collembolans and two replicates per set for group-reared collembolans. After 25 days of DEE, survival and reproduction were evaluated using the individually-reared collembolans. ELISA and RNA-Seq were performed using the group-reared collembolans as described for the first generation. The remaining collembolans that were fed in groups in the second generation were used to start the third generation. The procedure for the third-generation DEE was identical to that for the second except that there were three rather than two replicates per set for the group-reared collembolans.

### Survival and reproduction

The individually reared collembolans were used to evaluate the survival and reproduction of *F. candida* at the end of each generation. Several replicates were excluded from the analysis: (1) missing adult collembolans (five in BT treatment and five in CT treatment over three generations), which are assumed to have escaped because there was no corpse left; (2) eggs didn’t hatch due to overgrown fungi (six in BT treatment and five in CT treatment over three generations).

With the aid of a stereoscopic microscope (SMZ-10; Nikon, Minato, Japan), the number of living adults was determined as a measure of survival, and the number of juveniles produced was determined as a measure of reproduction. We calculated the mean number of juveniles produced per individually fed collembolan. Due to the wide variation in the reproduction data (7–41 juveniles per individually-reared collembolan), the data were log-transformed prior to conducting statistical analysis. Student’s *t*-test was used to analyze the differences between the two treatments in each generation.

In addition, the data for the reproduction of all BT and CT sets over three generations were subjected to a two-way ANOVA, followed by the least significant difference test, with diet treatment, “generation,” and their interaction as fixed factors. Generation is not an independent variable, but we used “generation” as a label representing unknown variable factors in order to test whether the reproductions of *F. candida* over three generations in the same treatment were affected by unpredictable environment stress. All of these statistical analyses were performed with IBM SPSS Statistics 24 (version R24.0.0.0). Differences were considered significant at *p* < 0.05.

### Enzyme-linked immunosorbent assay

The concentration of Cry1F in the diets and in the *F. candida* fed in groups was measured by ELISA for each replicate in each generation. The QuantiPlate Kit for Cry1F (Envirologix Inc., Portland, ME, USA) was used to detect Cry1F in a two- to four- mg sample of the fresh diet in each replicate, in a two- to four- mg sample of the diet in each replicate after 2 days of feeding, and in 20 individuals of *F. candida* (2–5 mg) per replicate. Before the test, all *F. candida* samples were washed in a PBST extraction buffer (phosphate-buffered saline with Tween-20, pH 7.4) to remove any Cry1F toxin from the outer surface of the collembolan cuticles. Then, all diet samples and *F. candida* samples were fully ground with an electric grinding rod and extracted with a PBST extraction buffer (phosphate-buffered saline with Tween-20, pH 7.4), respectively. ELISA was performed according to the manufacturer’s instructions. A two-way ANOVA was used to compare Cry1F concentrations of three BT sets over three generations by using the software IBM SPSS Statistics 24 (version R24.0.0.0). Differences were considered significant at *p* < 0.05.

### RNA extraction, sequencing, and unigene annotation

The miRNeasy® Mini Kit (Qiagen, Hilden, Germany) was used to extract the total RNA from 25–35 individuals per replicate of the collembolans that were fed in groups for each generation. The RNA concentration and integrity was evaluated with Aglient 2100 Bioanalyzer (Agilent, Santa Clara, CA, USA).

The cDNA library construction, Illumina sequencing, and de novo assembly of RNA samples were carried out by Hangzhou 1gene Technology Co., Ltd (Hangzhou, China). All cDNA libraries were constructed using the NEBNext®Ultra™ RNA Library Prep Kit for Illumina® (NEB, Ipswich, MA, USA) following the protocol described by the manufacturer. The libraries were sequenced with 150-bp paired-end reads on an Illumina Hiseq 4000 platform (San Diego, CA, USA). After sequencing, the raw reads (about 6 GB of data for each replicate) were filtered to remove adaptor sequences, duplication sequences, and low-quality sequences. The clean reads were de novo assembled into unigenes using Trinity and SOAPdenovo-Trans. Six public databases were used to annotate unigenes with BLASTx or BLASTn (*E*-value < 10^−5^), including NCBI Nr and Nt databases (http://www.ncbi.nlm.nih.gov/), SwissProt (http://www.expasy.ch/sprot/), KEGG (http://www.genome.jp/kegg/), COG (http://www.ncbi.nlm.nih.gov/COG/), and GO (http://www.geneontology.org/).

### Differential gene expression and biological marker expression

Based on strict synchronization and multiple repeats of samples, we screened DEGs between groups of CT and BT sets in the same generation. The expression levels of all unigenes were calculated using the fragments per kb per million fragments (FPKM) method: FPKM = (10^6^*C*)/(*NL*/10^3^). The data of two or three replicates for each set in every generation were averaged, and the whole transcriptome *gene expression* of all CT and BT sets were compared. DEGs were determined by the condition of (fold change, |log2 ratio| of FPKM (BT vs. CT)) ≥ 1 and false discovery rate ≤ 0.05. Co-expressed DEGs over three generations were screened and displayed with Venn diagrams. All DEGs over three generations were analyzed by heatmaps and the hierarchical clustering.

Additionally, we carefully checked the expression of seven known biological markers, whose changes in expression level are often used to indicate that the test organism is experiencing stress. These genes encode the following proteins: the antioxidant-related enzymes catalase (CAT) (Niu et al., 2017; Yousef, Abdelfattah & Augustyniak, 2017) and superoxide dismutase (SOD) (Yang et al., 2015; Yousef, Abdelfattah & Augustyniak, 2017), the detoxification-related enzymes glutathione S-transferase (GST) (Niu et al., 2017; Oliveira et al., 2015), carboxylesterase (CES) (Niu et al., 2017; Yang et al., 2015), and glutathione reductase (GR) (Yang et al., 2015), a metallothionein-like motif containing protein (MTC) (Nakamori & Kaneko, 2013), which is unique in *F. candida* and which is very sensitive to different heavy metal ions, and the heat shock protein 70 (HSP70) (Liu et al., 2010), which is sensitive to insecticides, drought, and other environmental stresses. The expression profiles of unigenes annotated to these biological markers were examined and their expression levels were compared among different samples. All these genes of the seven biological markers, over three generations, were analyzed by heatmaps and the hierarchical clustering.

The software R (R Core Team, 2017) was used for the heatmaps (R package “pheatmap” (Kolde, 2019)) and the hierarchical clustering (“dist” and “hclust” orders, distance method as “euclidean,” and cluster method as “complete”) of all DEGs and biological markers over three generations (expression data of DEGs were transformed by using the common logarithm of the counts plus 1).

## Results

### Cry1F concentrations in the diet and *F. candida*

Enzyme-linked immunosorbent assay measures showed that no Cry1F toxin was detected in the pure yeast diet or in the *F. candida* that were fed the CT diet. The average concentration (mean ± SE) of Cry1F was 415 ± 15 μg/g in the fresh BT diet and 358 ± 57 μg/g in the BT diet after 2 days of feeding, indicating that Cry1F was continually present in the experiment. The average concentrations of Cry1F in collembolans that were fed the BT diet were 3.5 ± 1.3, 2.7 ± 1.4, and 5.2 ± 1.0 μg/g at the end of the first, second, and third generation, respectively. These concentrations were not significantly different based on a two-way ANOVA (*p* = 0.429) and suggest that the Cry1F was continuously ingested by collembolans.

### Survival and reproduction of *F. candida*

**Figure 2:**
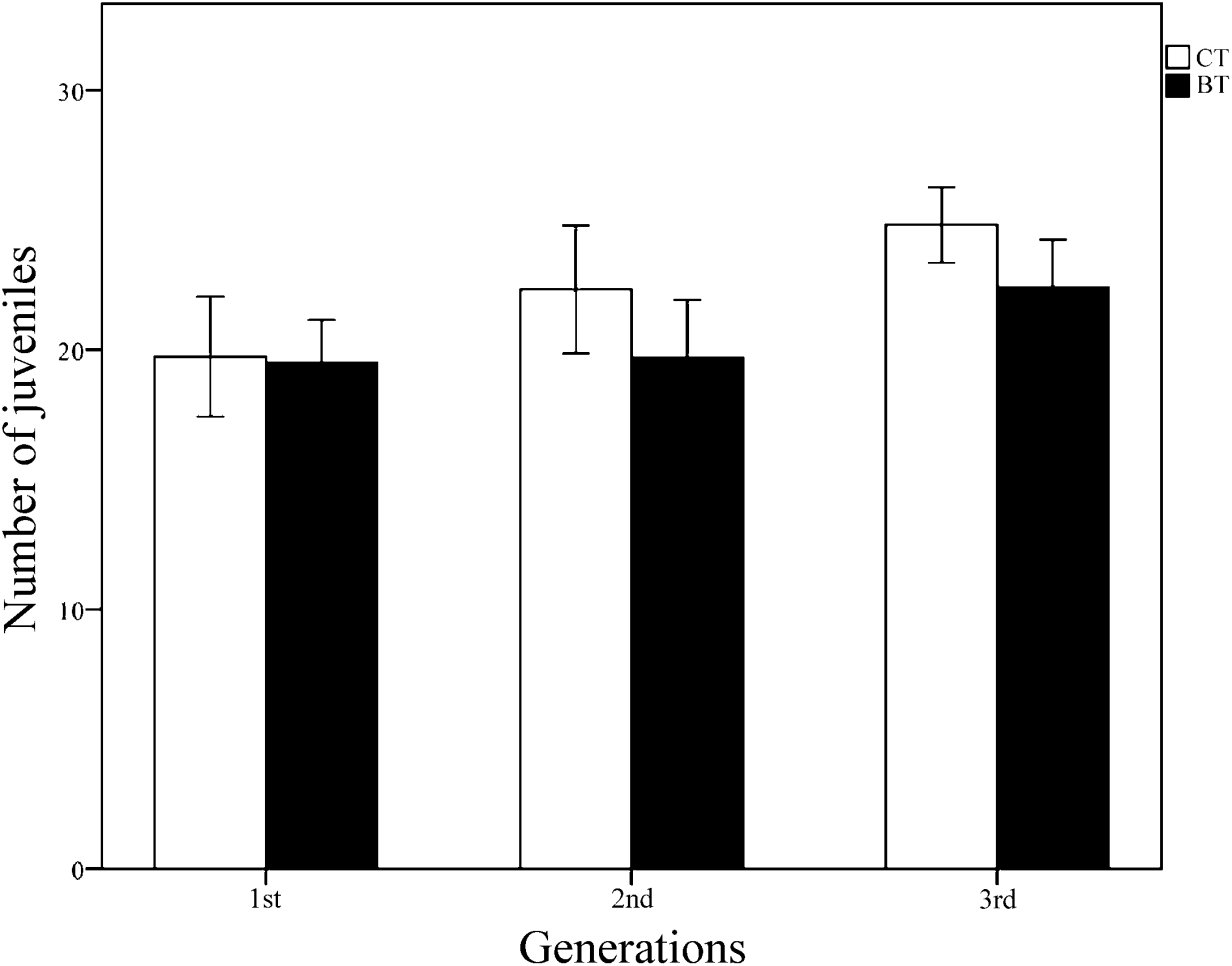
The reproduction (number of juveniles produced per individually fed collembolan) of three generations of *F. candida* as affected by addition of Cry1F to an artificial diet. The collembolans were individually fed pure yeast powder (CT set) or yeast powder + Cry1F protein (BT set). Values are means ± SE (*n* = 24–38). Across all generations, independent Student’s *t*-test between CT and BT sets for each generation indicate no significant difference, with *p* = 0.912, 0.114, 0.071 for the 1st, 2nd, and 3rd generations, respectively (significant difference *p* < 0.05).

All collembolans in both the CT and BT sets survived and reached the adult stage for all three generations, suggesting that Cry1F has no obvious negative effect on collembolan survival. Over the three generations, the mean (± SE) number of juveniles produced per individually fed collembolan was 22.57 ± 0.635 for 100 samples in the CT set and 20.70 ± 0.590 for 99 samples in the BT set. According to Student’s *t*-test for each generation, the number of juveniles produced per collembolan did not significantly differ between the CT and BT sets for any of the three generations (*p* = 0.912, 0.114, 0.071 for generations 1, 2 and 3, respectively) (Fig. 2). Two-way ANOVA (with diet treatment, “generation,” and their interaction as fixed factors, Table 1) showed that the mean number of juveniles produced per collembolan was significantly affected by “generation” (*p* = 0.001) but not by diet treatment (*p* = 0.068) or the interaction between diet treatment and “generation” (*p* = 0.410). The results suggest that even in the same treatment culture condition, the reproductions of *F. candida* were still affected by some unpredictable environment stress instead of the BT diet.

**Table 1:** A two-way ANOVA of reproduction^a^ with diet treatment, “generation,”^b^ and their interaction as fixed factors, followed by LSD test.

Source	SS	d*f*	MS	*F*	*p*-value
diet treatment	0.052	1	0.052	3.363	0.068
“generation”	0.227	2	0.114	7.414	0.001*
diet treatment × “generation”	0.027	2	0.014	0.896	0.410
error	2.956	193	0.015		
total	348.507	199			

**Notes:**

a The number of juveniles produced per collembolan in the individual feeding test was calculated as reproduction value.

b “generation” is a label representing unknow variable factors, in order to test whether the reproductions of *F. candida* over three generations in the same culture condition were still affected by unpredictable environment stress.

* *p* < 0.05.

### Transcriptome sequencing and annotation

A total of 316,693,647 raw reads for 16 samples were generated, and deposited in the NCBI Sequence Read Archive (SRP132745). After data filtering and de novo assembly, 284,174,422 clean reads were assembled into 93,976 unigenes. The total length of these unigenes was 155,829,628 bp. The mean length of the unigenes was 1,658 bp, and N50 length was 3,292 bp. The sequence data of each type of sample were unbiased, and enough reads were obtained to perform gene expression analyses. The quantity and quality of the RNA-Seq data are shown in Table 2.

**Table 2:** Summary of *F. candida* transcriptome sequencing and assembly for CT and BT sets over three generations.

Statistic	CT			BT		
	1st	2nd	3rd	1st	2nd	3rd
Raw reads	55,491,201	52,314,588	51,127,722	55,080,974	49,522,385	53,156,777
Clean reads	51,147,377	43,971,149	47,089,957	50,778,618	42,728,374	48,458,947
Q20 (%)	98.21	98.46	99.00	98.33	98.36	98.80
GC content (%)	40.63	42.93	42.96	41.97	43.63	43.84

Of the total number of unigenes, 55,390 (58.94%), 17,230 (18.33%), 47,734 (50.79%), 43,284 (46.06%), 31,016 (33.00%), and 18,603 (19.80%) were annotated in NCBI Nr, NCBI Nt, SwissProt, KEGG, COG, and GO databases, respectively. Overall, 57,758 (61.46%) unigenes were annotated to known protein/nucleotide sequences.

### Differentially expressed genes (DEGs) over three generations

**Figure 3:**
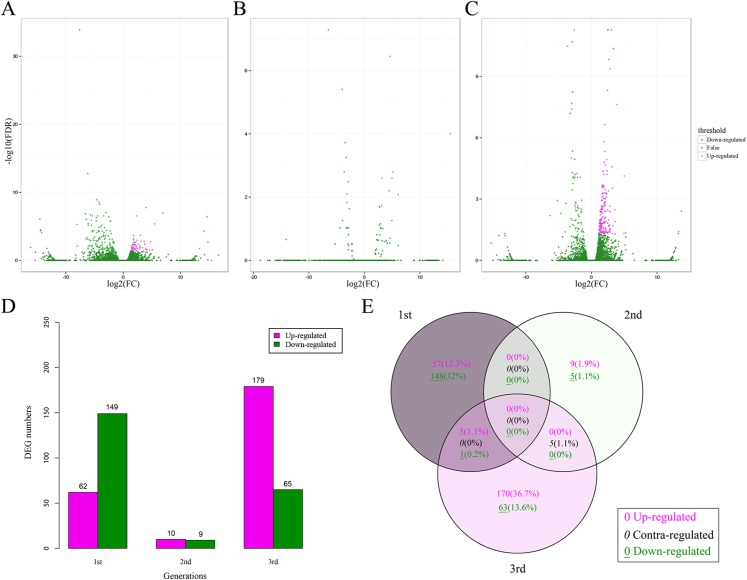
DEGs in *F. candida* fed diets with and without Cry1F over three generations. (A–C) Volcano plots of DEGs for the 1st, 2nd, and 3rd generation, respectively. DEGs: FC (|log2 ratio| of FPKM (BT vs. CT)) ≥ 1, FDR ≤ 0.05. (D) Numbers of up-regulated and down-regulated DEGs. (E) Venn diagrams of DEGs in three generations. DEGs, differentially expressed genes; FC, fold change; FPKM, fragments per kb per million fragments; FDR, false discovery rate.

A total of 463 DEGs (0.49% of all unigenes) between the CT and BT sets were identified for all three generations (Figs. 3A–3C), including 211, 19, and 244 DEGs for the first, second, and third generation, respectively (Fig. 3D). There was no consistent tendency for either the up-regulation or down-regulation of DEGs and there was no consistent co-expression of DEGs over the three generations (Fig. 3E) and only six DEGs (five up-regulated and one down-regulated) were identified in both the first and third generations. Five contra-regulated DEGs were detected in the second and third generations. The functions of these 11 DEGs are unknown.

**Figure 4:**
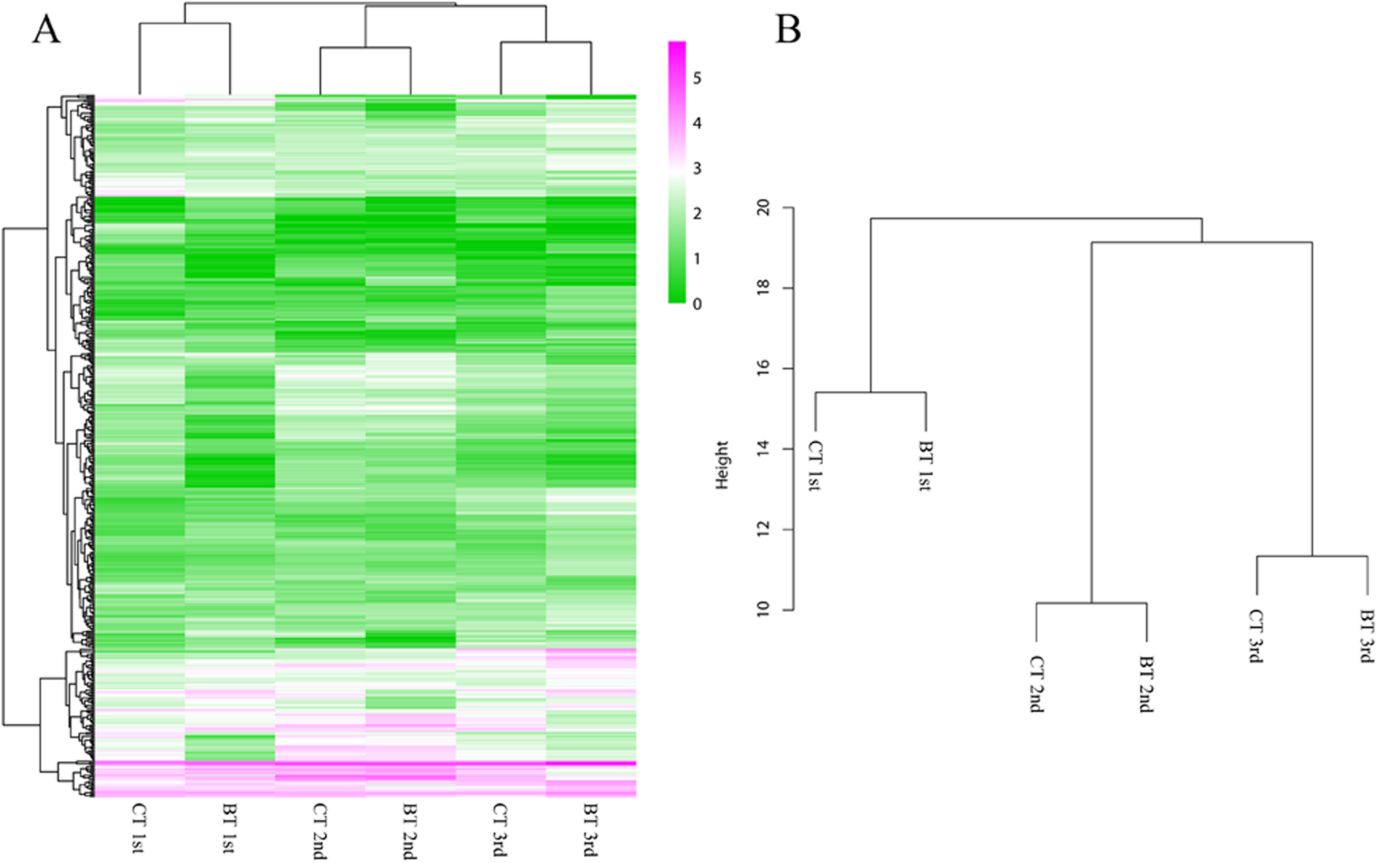
Heatmap and hierarchical clustering dendrogram of DEGs. (A) Heatmap of DEGs. Gene clusters were subjected to gene ontology analysis. Columns represent samples. The scale bar indicates log-transformed (common logarithm of the counts plus 1) gene expression values, with high expression depicted in red and low expression in green. (B) Hierarchical clustering dendrogram of six samples over three generation. Log-transformed data of DEGs were used for analysis. The distance method was set as “euclidean” and the cluster method was set as “complete”.

The heatmap analysis of DEGs over three generations showed similar expression patterns of the CT and BT sets in each generation (Fig. 4A). The hierarchical clustering of all samples demonstrated that the CT and BT samples of the same generation were always clustered together, instead of the CT or the BT sets of different generations (Fig. 4B).

### Biological marker expression profile

**Figure 5:**
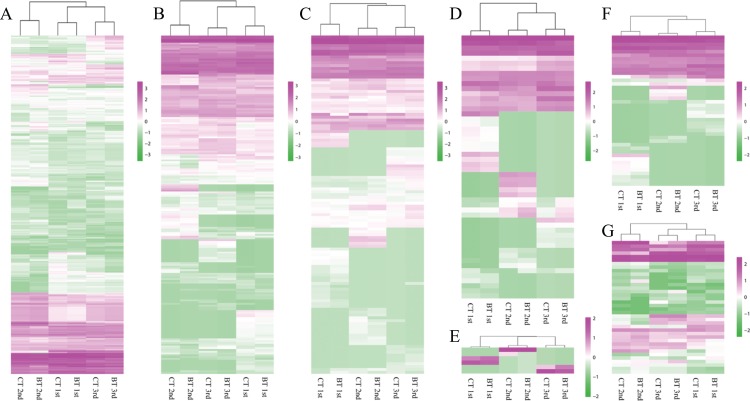
Heatmap of seven biomarkers. Gene clusters from hierarchical classification were subjected to gene ontology analysis. Columns represent samples. The scale bar indicates log-transformed gene expression values, with high expression depicted in red and low expression in green. (A) Carboxylesterase (CES). (B) Glutathione S-transferase (GST). (C) Heat shock protein 70 (HSP70). (D) Catalase (CAT). (E) Glutathione reductase (GR). (F) Superoxide dismutase (SOD). (G) Metallothionein-like motif containing protein (MTC).

A total of 855 unigenes for the seven biomarkers (CAT, GST, SOD, GR, CES, MTC, and HSP70) were annotated, but only five unigenes were differentially expressed between the CT and BT sets, that is, were up- or down-regulated by more than two-fold in the BT set (Table 3): one unigene of CAT (CL2174.Contig5_All) was up-regulated in the third generation, three unigenes of CES (Unigene7933_All, CL5034.Contig2_All, and CL6365.Contig1_All) encoding CES type B were up- or down-regulated in the first or the third generations, one unigene of HSP70 (Unigene15285_All) was greatly down-regulated, that is, there was a five-fold decrease in expression, in the first generation. However, the expression of the five DEGs was significantly different in the BT vs. CT sets in only a specific generation, and none of those changes are consistent in all three generations (Table 3). Furthermore, the gene expression hierarchical clustering of the seven markers (Fig. 5) clearly showed that the CT and BT sets from the same generation always clustered together, which confirms that Cry1F treatment did not affect the functions related to the seven biological markers.

**Table 3:** Summary of annotated unigenes and DEGs^a^ of seven biomarkers of *F. candida* over three generations.

Biomarker	Number of unigenes	Name of unigene	1st		2nd		3rd	
			FC^b^	FDR	FC	FDR	FC	FDR
CAT	52	CL2174.Contig5_All	0.443	1.000	1.859	1.000	2.857	0.042*
GST	191	–	–	–	–	–	–	–
SOD	42	–	–	–	–	–	–	–
GR	6	–	–	–	–	–	–	–
CES	408	Unigene7933_All	1.664	0.042*	−2.469	1.000	0.225	1.000
		CL5034.Contig2_All	−1.968	0.023*	0.366	1.000	−0.410	1.000
		CL6365.Contig1_All	0.794	1.000	−0.104	1.000	2.054	0.000*
MTC	38	–	–	–	–	–	–	–
HSP70	118	Unigene15285_All	−5.226	0.043*	−2.191	1.000	−2.341	1.000
SUM	855							

**Notes:**

DEGs, differentially expressed genes; FC, fold change; FDR, false discovery rate; FPKM, fragments per kb per million fragments; CAT, catalase; GST, glutathione S-transferase; SOD, superoxide dismutase; GR, glutathione reductase; CES, carboxylesterase; MTC, metallothionein-like motif containing protein; HSP70, heat shock protein 70.

a DEGs: |FC| ≥ 1, FDR ≤ 0.05.

b FC: log2 ratio of FPKM (BT vs. CT).

* FDR ≤ 0.05.

## Discussion

Multi-generational laboratory tests are useful for detecting the chronic toxicity of pollutants on collembolans (Campiche et al., 2007; van Gestel et al., 2017). Most previous assessments of *B. thuringiensis* toxins or crops on collembolans involved the assessment of short-term exposure (one generation) rather than chronic toxicity (Yang et al., 2015, 2018; Zhang et al., 2017a). With the long-term cultivation of *B. thuringiensis* crops, however, toxicity may be undetectable by the traditional one-generation risk assessment methods and a finite number of physiological indices, which may increase in subsequent generations because of continuous exposure to *B. thuringiensis* toxins. Therefore, it is necessary to test the effects of *B. thuringiensis* toxins on NTOs with multi-generational toxicological assessments. Two previous laboratory-based multi-generational risk assessments of *B. thuringiensis* toxins on collembolans (Bakonyi et al., 2011; Szabó, Seres & Bakonyi, 2017) found that feeding on leaves of *B. thuringiensis* maize (*MON 810*) affected egg production, growth rate, and food preference in *F. candida*. In addition, Yuan et al. (2011) found that feeding on *B. thuringiensis* rice decreased collembolan CAT activity. However, these results are difficult to explain (except to note that *B. thuringiensis* and non-*B. thuringiensis* plant tissues may differ in properties other than the presence or absence of the *B. thuringiensis* toxin). The current study used a diet that was identical except for the presence or absence of the *B. thuringiensis* toxin. That *F. candida* is parthenogenetic facilitated the synchronization of generations and the multi-generational assessment. We did not use a positive control, because most positive controls such as insecticides usually seriously affect the survival and the development of *F. candida*, making it hard to carry out multi-generational exposure experiments. It is challenging to find an appropriate positive control in a multi-generational study, to appropriately reduce the concentration of the insecticidal compounds, for new individuals for each generation to show exposure, or to find another species that can survive multiple generations with sub-lethal effects.

In addition to comparing *F. candida* survival and reproduction, the current study also considered the effects of the *B. thuringiensis* toxin on gene expression. Microarray-based detection methods have been widely used to evaluate the molecular effect of pollutants on test organisms (Baillon et al., 2016; Roelofs et al., 2009; Yuan et al., 2014). Compared with microarray, RNA-Seq is more comprehensive and is better able to detect a potential risk, since it does not rely on a pre-designed complement sequence detection probe it enables the identification of genetic variants and it can quantify and profile overall gene expression, including rare and novel transcripts (Montgomery et al., 2010; Sultan et al., 2008; Wang, Gerstein & Snyder, 2009). If the pollutants are harmful to *F. candida*, they should create a constant stress and result in the co-expression of DEGs at different exposure times (Qiao et al., 2015) but this was not the case in the current study. The expression pattern of DEGs was more similar between the CT and BT sets in the same generation than between the same diet treatment of different generations (Fig. 4) suggesting that the detected DEGs may be random events perhaps caused by uncontrolled conditions in the experiment rather than by Cry1F. Moreover, the statistical analysis showed that the *F. candida* reproduction significantly differed among generations but not between the diet treatments (Table 1), which further confirmed that Cry1F had no constant or cumulative effect on *F. candida*. Although we synchronized *F. candida* to stage before every generation and although we strictly controlled the culture conditions, there were probably some uncontrolled differences in season, diet quality, or other factors between generations.

The biological marker assay is commonly used for monitoring environmental contaminants because it is rapid, simple, and sensitive. Yousef, Abdelfattah & Augustyniak (2017) found that fertilizer industry pollutants greatly affected the activities of SOD and CAT in the grasshopper *Aiolopus thalassinus*. By assessing GST responses, Oliveira et al. (2015) assayed the risk of *F. candida* exposure to carbamazepine. Nakamori & Kaneko (2013) measured the effect of Cd exposure on *F. candida* by assessing the gene expression of MTC, which is a biomarker related to heavy metal detoxification. Most previous studies only determined the gene expression differences of one or several biomarkers by RT-qPCR. In our study, RNA-Seq provided a whole transcriptome profile and therefore, we were able to screen a large variety of important biomarker genes in *F. candida*, including some novel response genes.

## Conclusions

In this study, we established a laboratory-based multi-generational risk assessment of Cry1F for *F. candida*. In addition to assessing the effect of Cry1F on *F. candida* survival and reproduction, we also assessed the effect on gene expression by using a comparative transcriptome analysis for the first time. Our results demonstrated that Cry1F did not affect the survival or reproduction of *F. candida* over three consecutive generations and did not alter global gene expression levels or the expression profiles of seven sensitive biological markers. In summary, these data indicate that Cry1F does not have harmful effects on the non-target organism *F. candida*.

## Supplemental Information

10.7717/peerj.6924/supp-1 Supplemental Information 1 Raw data for the concentrations of Cry1F in the diets and in *F. candida* fed in groups measured by ELISA.

10.7717/peerj.6924/supp-2 Supplemental Information 2 Raw data for survival and reproduction tests.

## Additional Information and Declarations

### DNA Deposition

The following information was supplied regarding the deposition of DNA sequences: The RNA-Seq data is available in the NCBI Sequence Read Archive (SRP132745) and at https://www.ncbi.nlm.nih.gov/Traces/study/?acc=PRJNA433895&go=go.

### Data Availability

The following information was supplied regarding data availability: Raw measurements are available as Supplemental Files.
